# Barrierless Electron Transfer in a Photosynthetic Reaction Center Model

**DOI:** 10.1002/anie.202422633

**Published:** 2025-02-21

**Authors:** Tobias Ullrich, Ivana Ramírez‐Wierzbicki, Leonardo D. Slep, Alejandro Cadranel

**Affiliations:** ^1^ Department Chemie und Pharmazie Physikalische Chemie Friedrich-Alexander-Universität Erlangen-Nürnberg Egerlandstraße 3 91058 Erlangen Germany; ^2^ Friedrich-Alexander-Universität Erlangen-Nürnberg (FAU) Interdisciplinary Center for Molecular Materials Egerlandstr. 3 91058 Erlangen Germany; ^3^ Universidad de Buenos Aires Facultad de Ciencias Exactas y Naturales Departamento de Química Inorgánica Analítica y Química Física Pabellón 2 Ciudad Universitaria C1428EHA Buenos Aires; ^4^ CONICET – Universidad de Buenos Aires Instituto de Química Física de Materiales Medio Ambiente y Energía (INQUIMAE) Pabellón 2 Ciudad Universitaria C1428EHA Buenos Aires Argentina

**Keywords:** Class III, Cyanide Bridge, Electron Delocalization, Photoinduced Mixed Valence Systems, Ultrafast IR

## Abstract

Vibrational spectroscopy is the ultimate tool to reveal whether any donor‐acceptor system is truly delocalized and therefore characterized by a single‐welled potential energy surface, or if it is marginally localized with a barrier that defines two minima. Our femtosecond IR absorption investigations on cyanide‐bridged mixed valence systems show a broad, intense and downshifted CN vibration signature, revealing, for the first time, an asymmetric, fully delocalized Class III photoinduced mixed valence system.

## Graphical abstract

Ultrafast IR absorption spectroscopy was utilized to interrogate excited‐state electron transfer in a strongly‐coupled, cyanide‐bridged photoinduced mixed‐valent system. Despite structural asymmetry, a flat excited‐state potential energy surface with a single minimum was found, showing a Class III behavior. These findings were supported by DFT/TD‐DFT calculations.

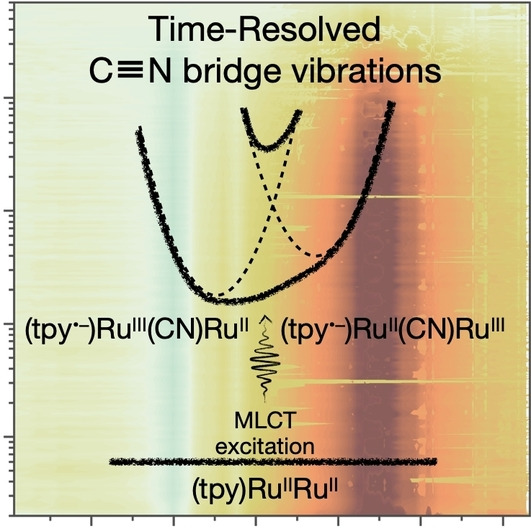

Solar‐energy conversion is based on careful management of photons and separated charges. In natural photosynthesis, photons are absorbed by the light‐harvesting complex and funneled to the photosynthetic reaction center (PRC), where primary charge separation occurs (Figure 1a). Nature manages the separated charges producing a photoinduced mixed‐valence (PI‐MV) system (Figure 1b), formed by the oxidized special pair of chlorophylls [Chl_1A_ ⋅ Chl_1B_]⋅^+^, and a radical anionic auxiliary chlorophyl Chl_2A_⋅^−^ (Figure 1a). Spatial location of Chl_2A_ close to Chl_1A_ favors an unbalanced distribution of the positive charge within [Chl_1A_ ⋅ Chl_1B_]⋅^+^, which, in turn, orients a hole‐transfer chain towards the water‐oxidation catalyst in photosystem II (PSII).[^1^, ^2^] The asymmetric location of the negative charge is determinant for solar fuel production. In this context, structurally asymmetric PI‐MV systems are the simplest models that most closely mimic this complex aspect of the PRC.^[3]^

One of the most sought‐after scenarios for electron transfer is full delocalization, interesting from a basic perspective but also for optoelectronics, solar energy conversion, and other applications were efficient charge management is imperative. Full delocalization is understood as the absence of thermal barriers for electron transfer, although this does not necessarily lead to a perfectly balanced 50–50 charge density distribution between the donor (D) and the acceptor (A), only attained in structurally symmetric systems.^[4]^ Notably, however, only few reports exist about fully delocalized PI‐MV systems, and they are all structurally symmetric.[^5^, ^6^, ^7^, ^8^, ^9^, ^10^, ^11^] This might obey the general consensus that electronic coupling maximizes, and, in consequence, the thermal barrier minimizes, in scenarios with minimal redox differences (zero for symmetric systems) between D and A. Furthermore, some chromophores undergoing photoinduced symmetry‐breaking charge separation^[12]^ which also produce fully delocalized PI‐MV systems,[^13^, ^14^, ^15^, ^16^, ^17^] also bear equivalent redox sites. In fact, fully delocalized mimics of the PRC based on structurally asymmetric PI‐MV systems have not been reported yet. This requires a fine control of the redox difference in the excited state.

Great efforts have been devoted to establish the presence or absence of thermal barriers for adiabatic electron transfer in the ground state, employing electroabsorption,^[18]^ optical spectroscopy[^19^, ^20^, ^21^] and (temperature‐dependent) EPR.[^22^, ^23^, ^24^, ^25^, ^26^] These techniques involve measurements of different physical phenomena which take place in different time frames.^[27]^ For example, solvent motions associated to electron transfer relax in some picoseconds, while vibrations at 2000 cm^−1^ correspond to a period of 17 fs. Thus, compounds with small barriers where electron transfer occurs in intermediate timescales feature solvent‐independent charge‐transfer absorptions, but vibrational signals differentiated according to each electron transfer isomer.^[28]^ This implies two minima in the potential energy surface (PES) corresponding to each isomer, and is known as Class II/III behavior.[^29^, ^30^] Barrierless electron transfer systems, known as Class III according to Robin and Day,^[31]^ are defined by a single‐minimum PES, and vibrational spectroscopy reveals the presence of only one species. While simple steady‐state FTIR experiments can determine the presence or absence of small barriers in strongly‐coupled donor‐acceptor systems,^[32]^ assessment of this question *in the excited state* requires ultrafast time‐resolved vibrational spectroscopy.

Cyanide‐bridged bimetallic PI‐MV systems are ideal candidates for the observation of barrierless electron transfer, because this bridge is known to promote strong electronic coupling,[^34^, ^35^, ^36^, ^37^, ^38^] and C≡N vibrations report on oxidation states of the redox‐active metal ions. Recently, MLCT excited states in bimetallic [Ru^II^(tpy)(bpy)(*μ*‐CN)Ru^II^(py)_4_(L)]^n+^ with L = ACN (**RuRuACN**) and DMAP (**RuRuDMAP**) (Figure 1c, tpy is 2,2’ : 6’,2”‐terpyridine, bpy is 2,2’‐bipyridine, py is pyridine, DMAP is 4‐dimethylaminopyridine) were investigated using femtosecond optical transient absorption spectroscopy (fsTAS). In both compounds, the dominating ^3^MLCTz excited states, with the excited hole sitting parallel to the intermetallic z axis, were identified as strongly‐coupled PI‐MV systems with intense PI‐IVCT bands.^[39]^ Reported herein is the exploration of these systems, together with the monometallic reference [Ru^II^(tpy)(bpy)(CN)]^+^ (**Ru**) (Figure 1c), using femtosecond IR absorption spectroscopy (fsIR). Results confirm that **RuRuACN** behaves similar like **Ru**, with the excited hole essentially localized on the {(tpy)Ru} fragment, while **RuRuDMAP** is fully delocalized, with a strongly mixed electronic configuration and no signs of any barrier for electron transfer in vibrational timescales, despite structural asymmetries. Small redox differences in the MLCT state of **RuRuDMAP** are achieved thanks to the excited electron on tpy⋅^−^, which destabilizes the metallic orbitals of the closest Ru ion.[^3^, ^39^, ^40^, ^41^]

**Figure 1:**
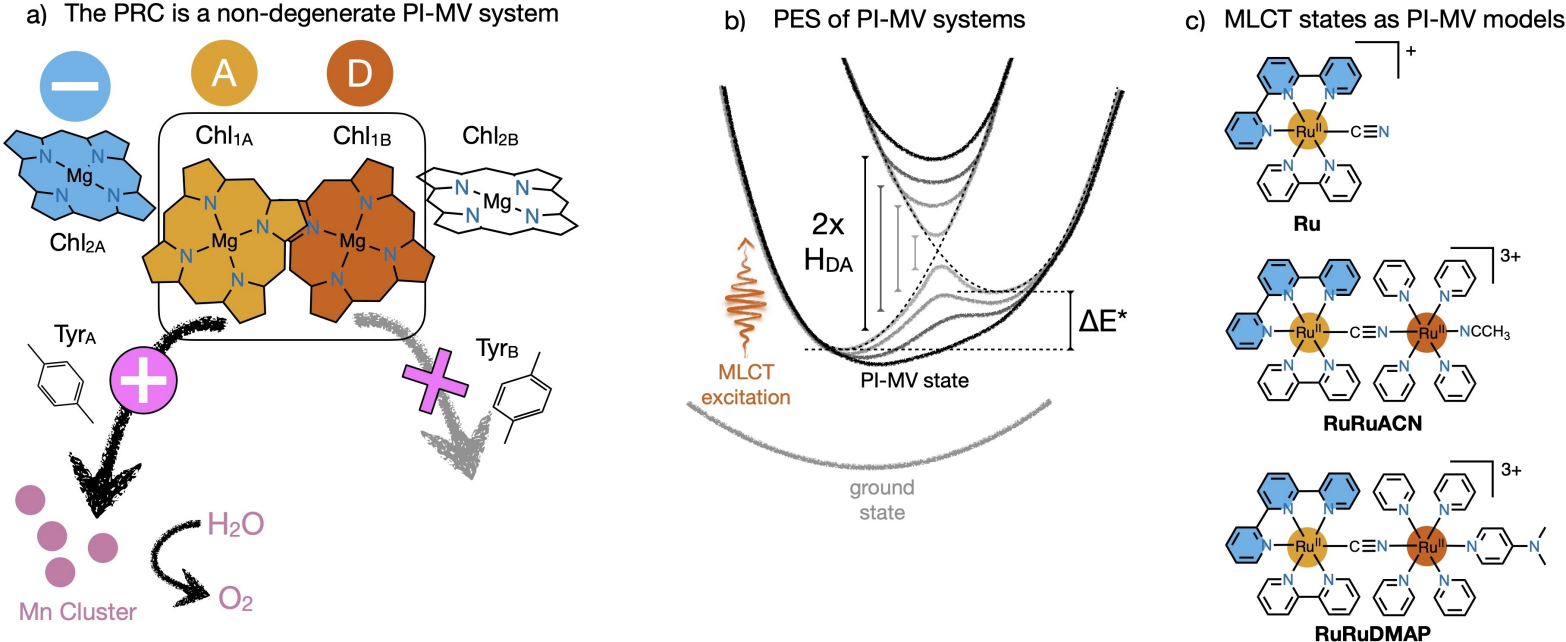
a) Scheme of the PRC in PSII, adapted from the crystal structure.^[33]^ b) Potential energy surfaces describing PI‐MV system. Highlighted are different extents of electronic coupling H_DA_, and the diabatic redox difference ΔE*. c) Monometallic reference **Ru** and donor‐acceptor bimetallic compounds **RuRuACN** and **RuRuDMAP**. Highlighted are the tpy ligand in blue, which is a radical anion in the MLCT state, the acceptor Ru ion in light orange, which is oxidized to Ru^III^ in the MLCT state, and, in the bimetallic compounds, the donor Ru ion in dark orange.

The electrochemical and spectroscopic properties of **Ru**, **RuRuACN** and **RuRuDMAP** have been discussed elsewhere.[^39^, ^42^] Their absorption spectra, shown in Figure S1, are dominated by MLCT absorptions in the visible, centered on {Ru(tpy)(bpy)}. Relevant data is collected in Table S1. Upon one‐electron oxidation, **RuRuACN** and **RuRuDMAP** show intense ground‐state IVCT absorptions in the NIR (Figure S2). In the excited state, optical transient absorption spectroscopy allowed to detect PI‐IVCT bands for the bimetallic compounds in acetonitrile. A broad band was observed for **RuRuACN** at 7900 cm^−1^, while that for **RuRuDMAP** at 6500 cm^−1^ was much narrower (Figure S3). In both cases, a poor solvent dependence was observed (Figure S3). Electronic couplings of 1320 and around 3200 cm^−1^, respectively, were derived.^[39]^

We started our vibrational investigations with **Ru** in acetonitrile. This solvent itself features a C≡N vibration at 2253 cm^−1^,^[43]^ precluding interferences with the 2000–2200 cm^−1^ region typical for inorganic cyanide C≡N vibration. In the ground state, the FTIR spectrum of **Ru** in acetonitrile features a sharp signal at 2088 cm^−1^ associated to C≡N stretching (Figure S3). Upon Ru^II^→Ru^III^ oxidation, this signal vanishes, and no new signals are observed (Figure S4). This process is electrochemically reversible. This behavior is consistent with a 98 % intensity decrease according to our theoretical calculations (see below, Table S2). Upon 480 nm excitation, a {(tpy⋅^−^)Ru^III^} ^3^MLCT excited state is populated.^[42]^ Using fsIR, a sharp negative signal peaking at 2088 cm^−1^ was observed, corresponding to ground‐state bleaching, accompanied by a broad and weak positive signal at 2065 cm^−1^ (Figure 2). No major spectral activity was noted, and, in fact, global analysis of the data afforded a monoexponential decay of 6.3 ns. This lifetime matched those derived from time‐resolved emission (Table S1). The species‐associated spectrum is shown in Figure 2.

**Figure 2:**
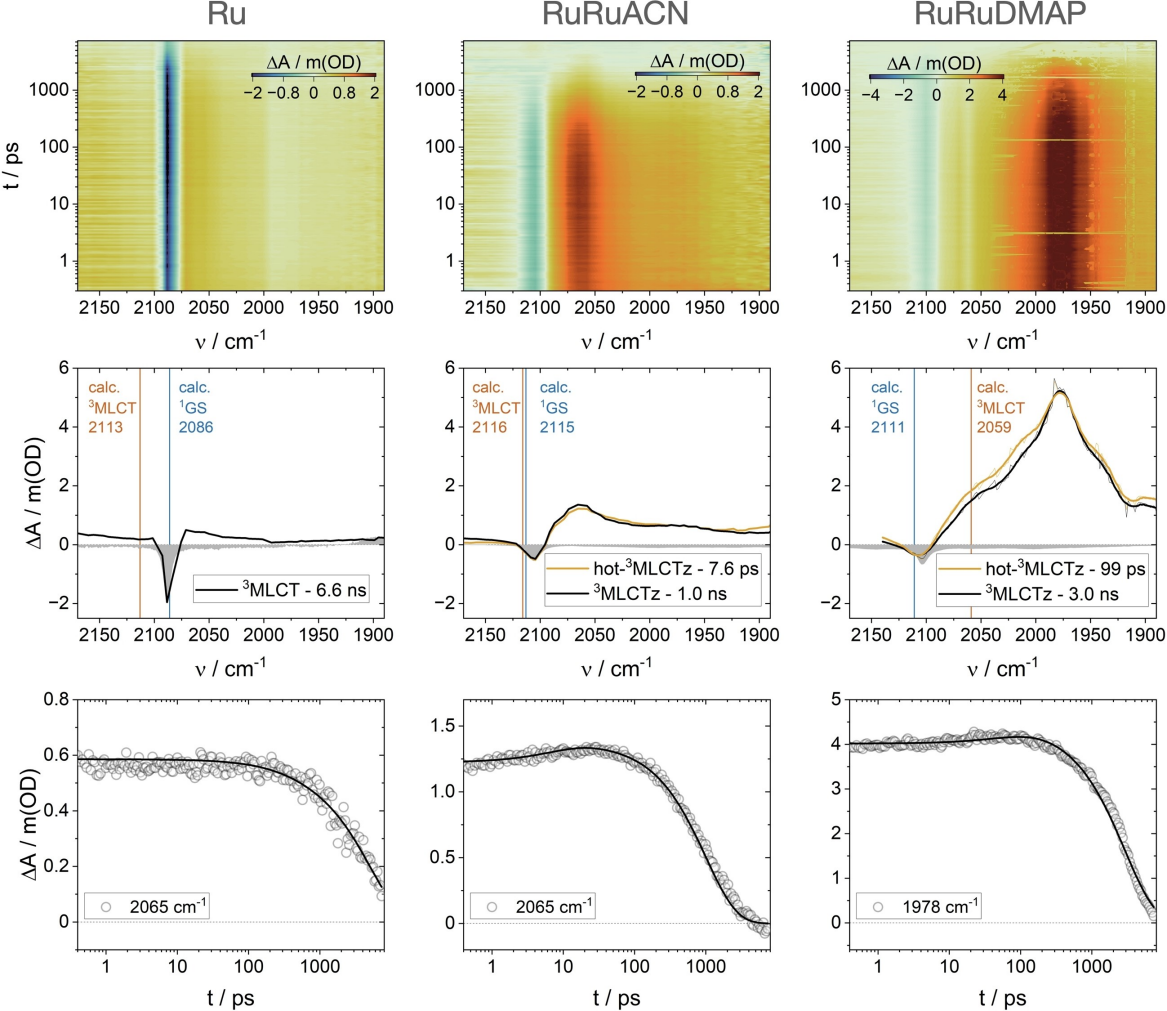
Top: Heatmap obtained by fsIR measurements on **Ru** (left), **RuRuACN** (center) and **RuRuDMAP** (right) under 480 nm excitation, in acetonitrile at room temperature. Middle: Species‐associated differential spectra obtained upon target analysis and calculated vibration frquencies. Bottom: Kinetic traces and fits at selected wavenumbers.

Next, we studied **RuRuACN**. In the ground‐state, the sharp C≡N stretching appears at 2105 cm^−1^, and upon oxidation, it downshifts to 2022 cm^−1^ (Figure S4), similar to other cyanide bridges.[^44^, ^45^, ^46^, ^47^] This has been explained as a consequence of a diminished backbonding from the newly oxidized N‐bound metal ion.^[44]^ Upon 480 nm excitation, a {(tpy⋅^−^)Ru^III^Ru^II^} ^3^MLCT excited state is populated.^[39]^ fsIR affords a mild negative signal associated to ground‐state bleaching and observed at 2105 cm^−1^, together with a positive band at 2065 cm^−1^ (Figure 2). Minor spectral activity without any energy shifts was well fitted with a biexponential decay with lifetimes of 7.6 ps and 1.0 ns. This was interpreted in terms of ^3^MLCTz population (where the excited hole lies in orbitals parallel to the z direction i.e. the intermetallic axis)[^41^, ^48^] that undergoes vibrational relaxation (VR) in few picoseconds and then decays to the ground state in the nanosecond timescale. Nicely, this matches the ^3^MLCTz lifetime obtained from optical transient absorption and time‐resolved emission.^[39]^ Important is to note that a minor deactivation channel observed before, involving the population of ^3^MLCTxy excited states (where the excited hole lies in orbitals perpendicular to the intermetallic axis), was not observed here. The spectral resemblance of the excited‐state vibrational absorptions between **Ru** and **RuRuACN** points to a similar electronic configuration. This is supported by previous calculations which suggested a totally unbalanced distribution of the positive charge density, and an electronic configuration where the excited hole sits mainly on {(tpy)Ru}.^[39]^

Finally, we moved to **RuRuDMAP**. In the ground state, the C≡N stretching is observed at 2104 cm^−1^ (Figure S4). Upon oxidation, it downshifts to 2038 cm^−1^ (Figure S4), like **RuRuACN**. However, the fsIR signals of the {(tpy⋅^−^)Ru^III^Ru^II^} ^3^MLCT state populated upon 480 nm excitation were different. A rather weak ground‐state bleaching at 2104 cm^−1^ was accompanied by a very broad and intense positive signal at 1978 cm^−1^ (Figure 2). Also in this case, only minor spectral activity was detected, and global analysis yielded a biexponential decay with lifetimes of 99 ps and 3.0 ns. This was also understood as ^3^MLCTz population that vibrationally relaxes in some picoseconds and then decays to the ground state in the nanosecond timescale. Here the vibrational relaxation times are somewhat longer than usual, but the absence of marked spectral shifts hinders other interpretations for this process. This implies inefficient vibrational energy transfer to intramolecular and solvent modes, which usually drive vibrational relaxation, probably due to electron delocalization and a specially flat PES. The nanosecond lifetime undoubtedly corresponds to the relaxed ^3^MLCTz, with a lifetime that matches those obtained from time‐resolved emission and optical transient absorption.^[39]^ The IR spectroscopy of ^3^MLCTz in **RuRuDMAP** is remarkable. The intensity and broadness, together with the pronounced downshift to 1978 cm^−1^ of the cyanide vibration signal reveal a strongly‐mixed PI‐MV system, where nuclear and electronic coordinates are entangled.[^49^, ^50^] C≡N stretchings of cyanide‐bridged Class III systems typically appear at 1970–1980 cm^−1^ for structurally symmetric,[^22^, ^51^] and 2000–2020 cm^−1^ for structurally asymmetric systems.[^52^, ^53^] Additionally, the 85 cm^−1^ FWHM of the IR band observed here surpasses the 65 cm^−1^ for a degenerate Class III system.^[51]^ It is worth to note that these FWHM values should be taken as qualitative indicators, since axial rotational conformers,[^54^, ^55^, ^56^] not specifically analyzed herein, and also low‐energy transitions of intraconfigurational (t_2g_→t_2g_) electronic origin,[^28^, ^29^, ^57^] might contribute to broaden the observed signals. However, most of the reported cyanide‐bridged mixed‐valence systems include these contributions, so FWHM values are still valuable metrics in comparative analyses. Thus, **RuRuDMAP** showcases the strongest red shift and widest vibrational absorption reported to date for a structurally asymmetric Class III system.

An excellent coincidence between the experimental electronic absorption spectroscopy of the ground and excited states and DFT and TD‐DFT computations on the lowest singlet and lowest triplet states of **Ru**, **RuRuACN** and **RuRuDMAP** has been shown.^[39]^ Herein, we analyzed the same outputs in terms of IR vibrations. In the ground state, calculated C≡N vibration frequencies perfectly match the experimental values (Figure 2), for the parent and one‐electron oxidized forms (Table S2). In the excited state, the trend observed experimentally is fully reproduced by the calculations, with essentially equivalent frequencies of **Ru** and **RuRuACN**, and a down‐shifted and very intense vibration for **RuRuDMAP**. However, the quantitative agreement is less accurate compared to that observed for the GS, probably due to the level of theory involved in the computations. Like the experiment, computations afford a remarkable enhancement of the C≡N stretching band intensity in the excited state, relative to the ground state, for **RuRuDMAP**, but not for **RuCN** and **RuRuACN** (Table S2).

An additional observation deserves attention. None of the compounds studied here displayed any significant spectral reshaping nor shifts in their IR absorption profiles in the 1950–2150 cm^−1^ region in acetonitrile during the observed time window of 0.4 ps–7 ns. This indicates no changes in excited‐state electronic configurations during the observed time window starting at around 150 fs after light absorption. For **Ru**, 480 nm excitation produces a {(tpy⋅^−^)Ru^III^} MLCT state that decays trivially since no electron donor is present. For the bimetallic compounds, it is useful to consider a Mulliken‐Hush picture of the ^3^MLCTz state (Figure 3). For strongly‐coupled systems undergoing adiabatic electron transfer, the barrier height depends on D−A electronic coupling H_DA_, the redox difference ΔE between the diabatic states and the reorganization energy λ. Equations (1) and (2) are the excited‐state versions of the well‐known equations for ground‐state systems,[^58^, ^59^, ^60^] derived for PI‐MV systems where charge‐transfer excitation creates, for example, a transiently oxidized A fragment and a transiently reduced charge‐transfer counterpart like tpy⋅^−^. There, ΔE* is the excited‐state diabatic redox difference, E_D_ is the redox potential of the donor, E_A_* is the excited‐state redox potential of the acceptor, E_red_ is the reduction potential of the charge‐transfer counterpart, E_00_* is the energy of the charge‐transfer excited state, E_PI‐IVCT_ is the energy of the PI‐IVCT band and λ* is the excited‐state reorganization energy.
(1)

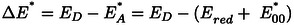



(2)

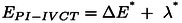




**Figure 3:**
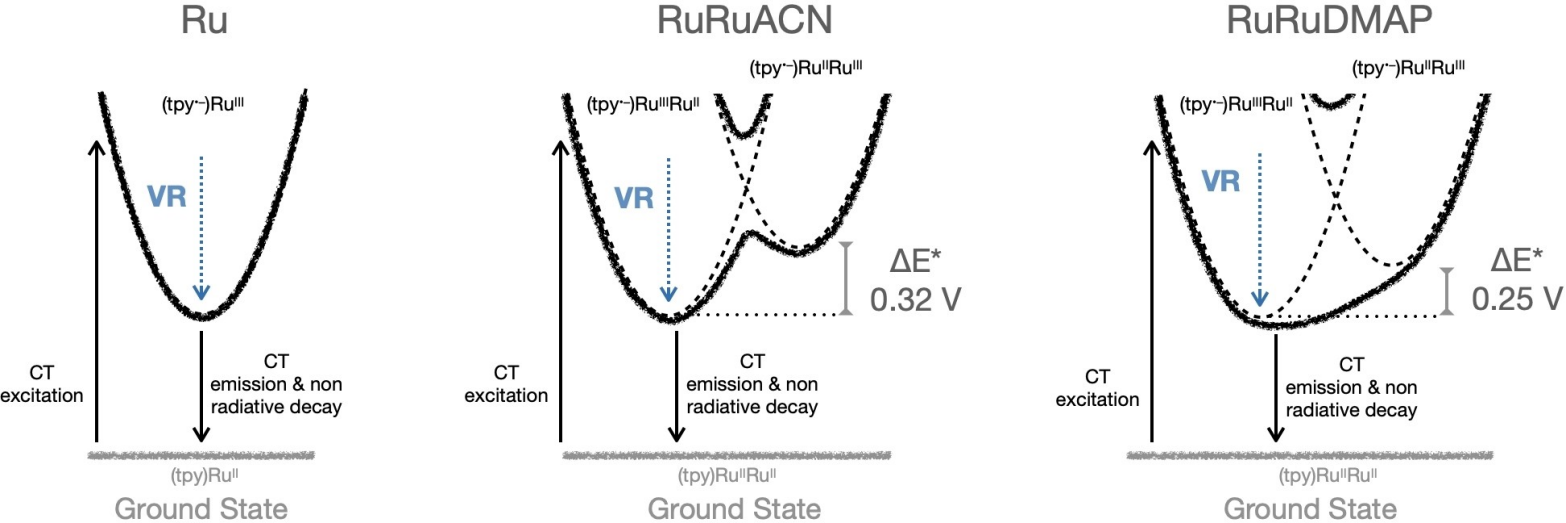
Excited state deactivation of **Ru**, **RuRuACN** and **RuRuDMAP** under a Mulliken‐Hush picture of the MLCT states.

For **RuRuACN**, 480 nm absorption mainly excites the {Ru(tpy)(bpy)} fragment. This generates MLCT states resembling those for **Ru**, with the excited hole mainly in {Ru(tpy)(bpy)}. For **RuRuACN** in the excited state, electronic coupling H_DA_ is 1230 cm^−1^ (0.16 eV), small compared to a reorganization energy of 0.66 eV according to equation (2). In this Class II PI‐MV system, the initially‐prepared MLCT state is similar to the no‐mixing situation represented by the diabatic PES. By virtue of a ΔE* of 0.32 eV, the initially‐prepared MLCT state is the most stable PI‐MV redox isomer. Therefore, no electron transfer processes within this PI‐MV systems are expected, no changes in the IR absorption spectra are observed, and it decays trivially to the ground state, like in the monometallic reference. For **RuRuDMAP**, 480 nm absorption also excites the {Ru(tpy)(bpy)} fragment, but the monomer‐like excited state is not observed. Simply, any other minimum visited during the decay cascade in timescales slower than tens of femtoseconds would have resulted in a different C≡N stretching signature, which was absent. This indicates that any internal conversion from the monomer‐like state to the fully‐delocalized single‐welled state is faster than 200 fs. In fact, H_DA_ is much higher with around 3200 cm^−1^ (0.40 eV), thanks to a smaller excited‐state redox difference of 0.25 eV. Here, H_DA_ is greater than half of the reorganization energy of only 0.56 eV. This is a strong indication of a barrierless Class III scenario. This confirms the first observation of a barrierless electron transfer in asymmetric PI‐MV systems.

It is worth to note that moving from **RuRuACN** to **RuRuDMAP** shrinks ΔE* from 0.32 to 0.25 eV. This redox difference is far from negligible, yet it affords a barrierless electron transfer in acetonitrile. This demonstrates that significant redox differences between D and A are compatible with full delocalization under strong electronic coupling.

In an attempt to induce localization in **RuRuDMAP**, solvent‐dependent fsIR studies were conducted. In symmetric PI‐MV systems like those generated in D−A−D or A−D−A systems upon photoinduced symmetry‐breaking charge transfer, less polar solvents favor more delocalized configurations because delocalization results in smaller dipolar moments.[^13^, ^14^, ^15^, ^16^, ^17^] In contrast, in the A−D−D’ systems explored here, delocalization to the second Ru ion (D’) increases the dipolar moment versus a situation more localized in the Ru(tpy) ion (D). Therefore, in polar solvents like acetonitrile, (*ϵ*=37.5) delocalized systems are favored, which matches our observations. In less polar solvents like acetone (*ϵ*=20.7) and dichloromethane (*ϵ*=9.1), localization is favored. In fact, in both acetone and dichloromethane, fsIR absorption spectra feature a coalescence of two different C≡N stretching signals at 2025 and 1980 cm^−1^, resulting in a rather flat maximum (Figure S5). These observations suggest a double‐welled PES. Early spectra display a maximum around 2025 cm^−1^, and late spectra resemble that one observed in acetonitrile with a signal around 1980 cm^−1^. Only marginal changes of the relative intensities occur during the observed time window, with lifetimes of 41 and 88 ps in acetone and dichloromethane, respectively. Therefore, these processes are probably mainly vibrational cooling, although some electron transfer character might also be involved. Importantly, this interpretation should be taken with care, since the 1980 cm^−1^ signal dominating in the three solvents at long time delays, hallmark of a delocalized state and a single‐minimum PES, appears too low‐shifted for a localized state. Thus, it is also possible that the 2025 cm^−1^ shoulder has intraconfigurational (t_2g_→t_2g_) electronic origin and that **RuRuDMAP** is also Class III in acetone and dichloromethane.

## Supporting Information

The authors have cited additional references within the Supporting Information.[^61^, ^62^, ^63^, ^64^]

## Conflict of Interests

The authors declare no conflict of interest.

## Supporting information

As a service to our authors and readers, this journal provides supporting information supplied by the authors. Such materials are peer reviewed and may be re‐organized for online delivery, but are not copy‐edited or typeset. Technical support issues arising from supporting information (other than missing files) should be addressed to the authors.

Supporting Information

## Data Availability

The data that support the findings of this study are available from the corresponding author upon reasonable request.
