# Supplementary material for: Barrierless Electron Transfer in a Photosynthetic Reaction Center Model
Source: Angew Chem Int Ed Engl. 2025 Feb 21;64(12):e202422633. doi: 10.1002/anie.202422633 (PMC11914954; doi:10.1002/anie.202422633)
Supplement: Supplementary file 1 — Supporting Information [file ANIE-64-e202422633-s001.pdf]

Angewandte Chemie
Eine Zeitschrift der Gesellschaft Deutscher Chemiker

Supporting Information

Barrierless Electron Transfer in a Photosynthetic Reaction Center Model

*T. Ullrich, I. Ramírez-Wierzbicki, L. D. Slep, A. Cadranel**

Supplementary Information
for
Barrierless electron transfer in a photosynthetic reaction center model

Tobias Ullrich,[a,b] Ivana Ramírez-Wierzbicki, [c,d] Leonardo D. Slep, [c,d] Alejandro Cadranel* [a,b,c,d]

[a] Department Chemie und Pharmazie, Physikalische Chemie Friedrich-Alexander-Universität Erlangen-Nürnberg, Egerlandstraße 3, 91058 Erlangen, Germany
[b] Friedrich-Alexander-Universität Erlangen-Nürnberg (FAU), Interdisciplinary Center for Molecular Materials, Egerlandstr. 3, 91058, Erlangen, Germany.
[c] Universidad de Buenos Aires, Facultad de Ciencias Exactas y Naturales, Departamento de Química Inorgánica, Analítica y Química Física, Pabellón 2, Ciudad Universitaria, C1428EHA, Buenos Aires, Argentina.
[d] CONICET – Universidad de Buenos Aires. Instituto de Química Física de Materiales, Medio Ambiente y Energía (INQUIMAE), Pabellón 2, Ciudad Universitaria, C1428EHA, Buenos Aires, Argentina.

*ale.cadranel@fau.de

Table of Contents

Experimental Details 2
Computational Details 2
Steady-State Electronic Absorption Spectroscopy 3
Figure S1........ 3
Figure S2........ 3
Optical Transient-Absorption Spectroscopy 4
Figure S3........ 4
Steady-State Vibrational Spectroscopy 4
Figure S4........ 4
Physicochemical properties 5
Table S1 5
Calculation of vibrational frequencies 5
Table S2 5
fsIR spectroscopy in different solvents 6
Figure S5........ 6
References 7

Experimental Details

RuCN and **RuRuL** were available from previous studies.[1,2]

Steady-state absorption was performed using a Shimadzu UV-1900i UV-Vis double-beam spectrometer (190 to 1100 nm). FTIR measurements were performed in a Shimadzu Prestige 21. IR spectroelectrochemistry was performed using a FRA 2 uAutolab Type III potentiostat and an OTTLE cell from Spectroelectrochemistry Reading, an improved model of the original version,[3] featuring CaF_2 windows, a Pt mesh as the working electrode, a second Pt mesh as the counter electrode, and a Ag foil as the reference electrode.

Femtosecond transient-absorption experiments with an IR probe (fsIR) were performed employing an Astrella-F-1K amplified Ti:sapphire femtosecond laser system from Coherent, operating at a repetition rate of 1kHz, with an 800 nm output and pulse duration of 80 fs, reaching a power of 5.5 W (5 mJ pulse energy). A fraction of 1.2 mJ was used for pump beam generation by a Topas Prime with standard NirUVis extension module from Light Conversion. Another fraction of 3.0 mJ, utilized as a probe beam, was guided through a delay line from Ultrafast Inc., providing a temporal delays between pump and probe beam of 8 ns. After the delay line the probe beam was used for IR pulse generation (~ 2.6-11 µm) by a Topas Prime with nDFG extension module from Light Conversion. Both visible pump and IR probe beam were guided into a commercial Helios IR spectrometer from Ultrafast Inc. Inside the transient IR spectrometer the IR probe pulse was divided in a reference and probe beam, which were detected by nitrogen cooled 32 x 2 pixel MCT detector. All measurements were conducted in a translating home-built cell with CaF_2 windows and a Teflon spacer of 250 μm. Pump energy was typically 3000 nJ, and a depolarizer was placed in the pump beam to avoid rotational dynamics. To analyze transient absorption data, we used a suggested procedure.[4] We started with SVD and global analysis, using an all-sequential decay model that provides evolution associated spectra of potentially intervening species, to determine the number of decaying species that participate in the decay cascade. However, this doesn't necessary yield differential spectra with genuine physicochemical meaning. Afterwards, a target analysis is applied, using specific target models that result in species associated spectra with true physicochemical meaning. Obtained data were treated by SVD, global and target analyses using the R- package TIMP and GloTarAn.[4–6] The instrument response function (IRF) and dispersion (chirp of the white light pulse) were modelled and taken into account during the fitting procedure.

Computational Details

Geometry optimizations of the singlet ground states of the different species and their respective lowest energy excited triplet states were performed with density functional theory. The computations were performed with Gaussian 09 using the B3LYP hybrid functional and the effective core potential basis set LanL2DZ as implemented in G09 . Solvation effects (acetonitrile) were taken into account employing the polarizable conductor continuum model (CPCM). The nature of the stationary points obtained in the optimization steps was confirmed in all cases by means of vibrational analyses.

Steady-State Electronic Absorption Spectroscopy

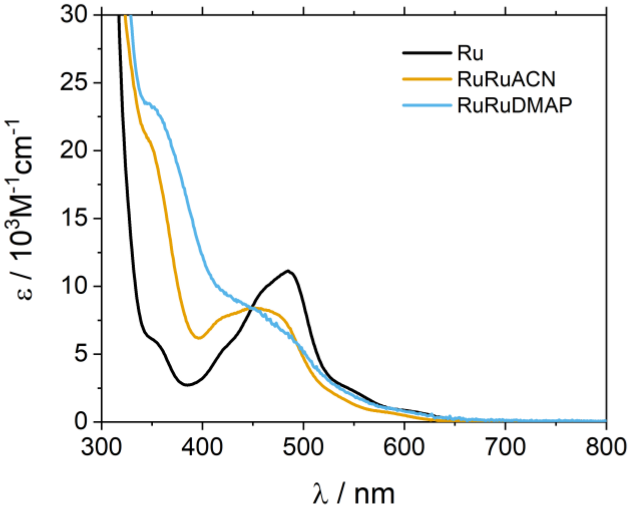

Figure S1. Absorption spectra of **Ru**, **RuRuACN** and **RuRuDMAP** in acetonitrile at room temperature.

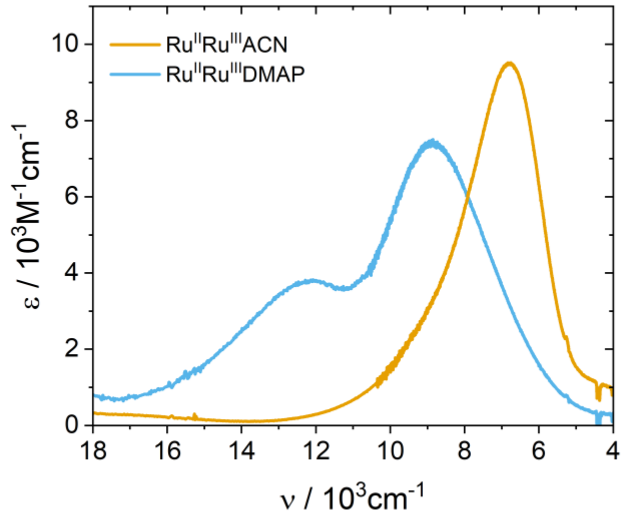

Figure S2. Ground-state IVCT absorptions of the one-electron oxidized forms of **RuRuACN** and **RuRuDMAP** in acetonitrile at room temperature. From reference [2]

Optical Transient-Absorption Spectroscopy

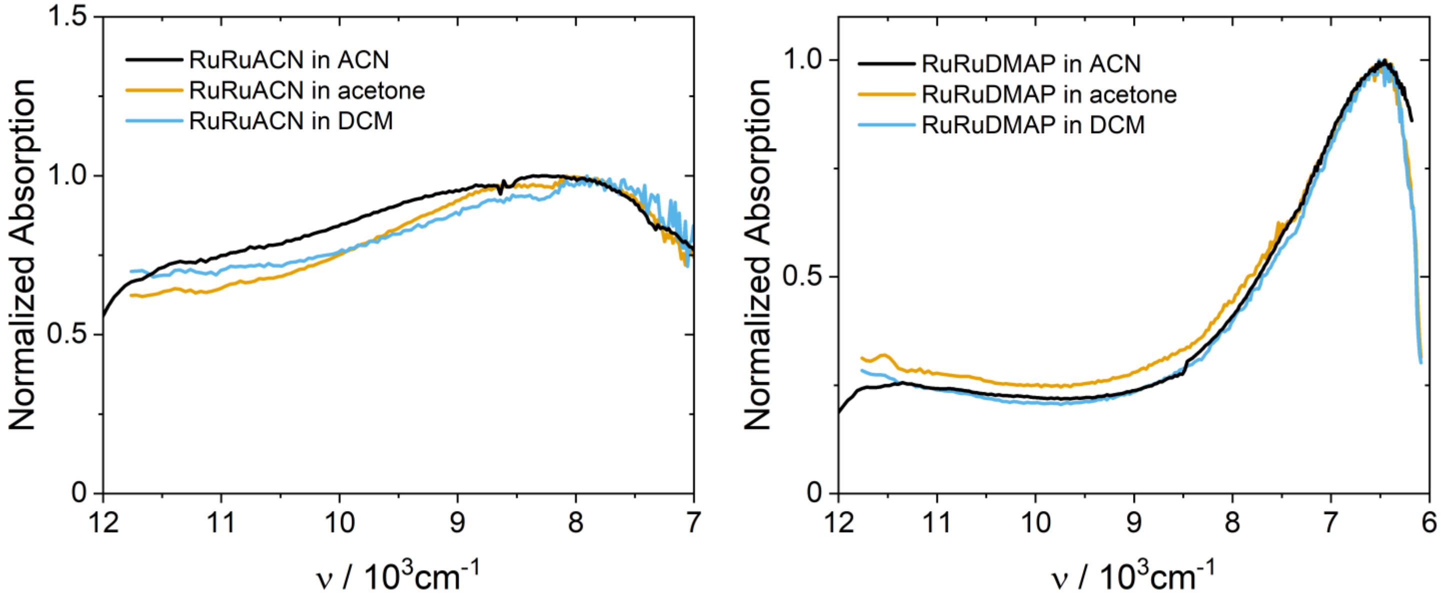

Figure S3. PI-IVCT bands of **RuRuACN** (left) and **RuRuDMAP** (right) in acetonitrile, acetone and dichloromethane at room temperature, measured using femtosecond optical transient absorption spectroscopy.

Steady-State Vibrational Spectroscopy

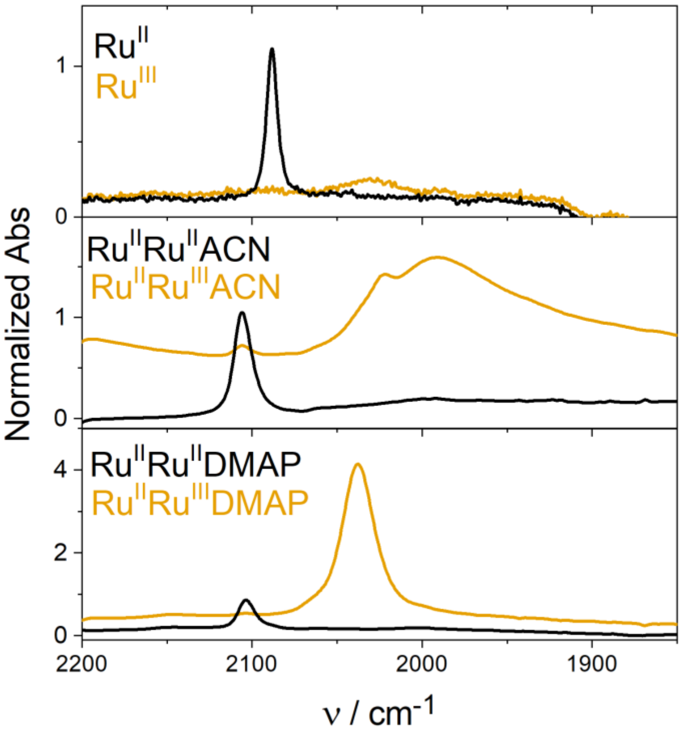

Figure S4. FTIR spectra of the parent (black) and one-electron oxidized (orange) forms of **Ru**, **RuRuACN** and **RuRuDMAP** in acetonitrile at room temperature. From reference [2]

Physicochemical properties

Table S1. Physicochemical properties of **Ru**, **RuRuACN** and **RuRuDMAP** in acetonitrile at room temperature. Extracted from references [1,2]

Compound	E / V vs Ag/AgCl		Absorption	Emission	
	Ru_{tb}	Ru_{py}	ν_{abs} / 10^3 cm^{-1} (ε / 10^3 M^{-1} cm^{-1})	ν_{em} / 10^3 cm^{-1} (Φ_{em} x10^{-3})	Dominant τ / ns
**Ru**	1.18	-	22.9 (8.5) (h)	14.7 (0.1)	5.9
**RuRuACN**	1.68	1.24	21.4 (8.1)<br>23.6 (7.8) (h)	14.7 (0.3)	9.4
**RuRuDMAP**	1.60	0.99	22.2 (h)	14.3 (0.3)	6.5

Calculation of vibrational frequencies

Table S2. Experimental C≡N vibrational frequencies and corresponding calculated values extracted from reference [2] and calculations reported in [7]

Compound		$\nu_{C≡N}$ / cm^{-1} (intensity / a.u.)					
		Ground State		1-e^- oxidized		^{3}MLCT state	
		calc	exp	calc	exp	calc	exp
**RuCN**		2086 (273)	2088	2124 (7)	n.d.	2113 (38)	2065
**RuRuACN**	bridge	2115 (386)	2105	2015 (7219)	2022	2116 (420)	2065
	ACN	2308 (45)	n.d.	2326 (4)	n.d.	2315 (30)	n.d.
**RuRuDMAP**		2111 (299)	2104	2052 (4123)	2037	2059 (6041)	1978

fsIR spectroscopy in different solvents

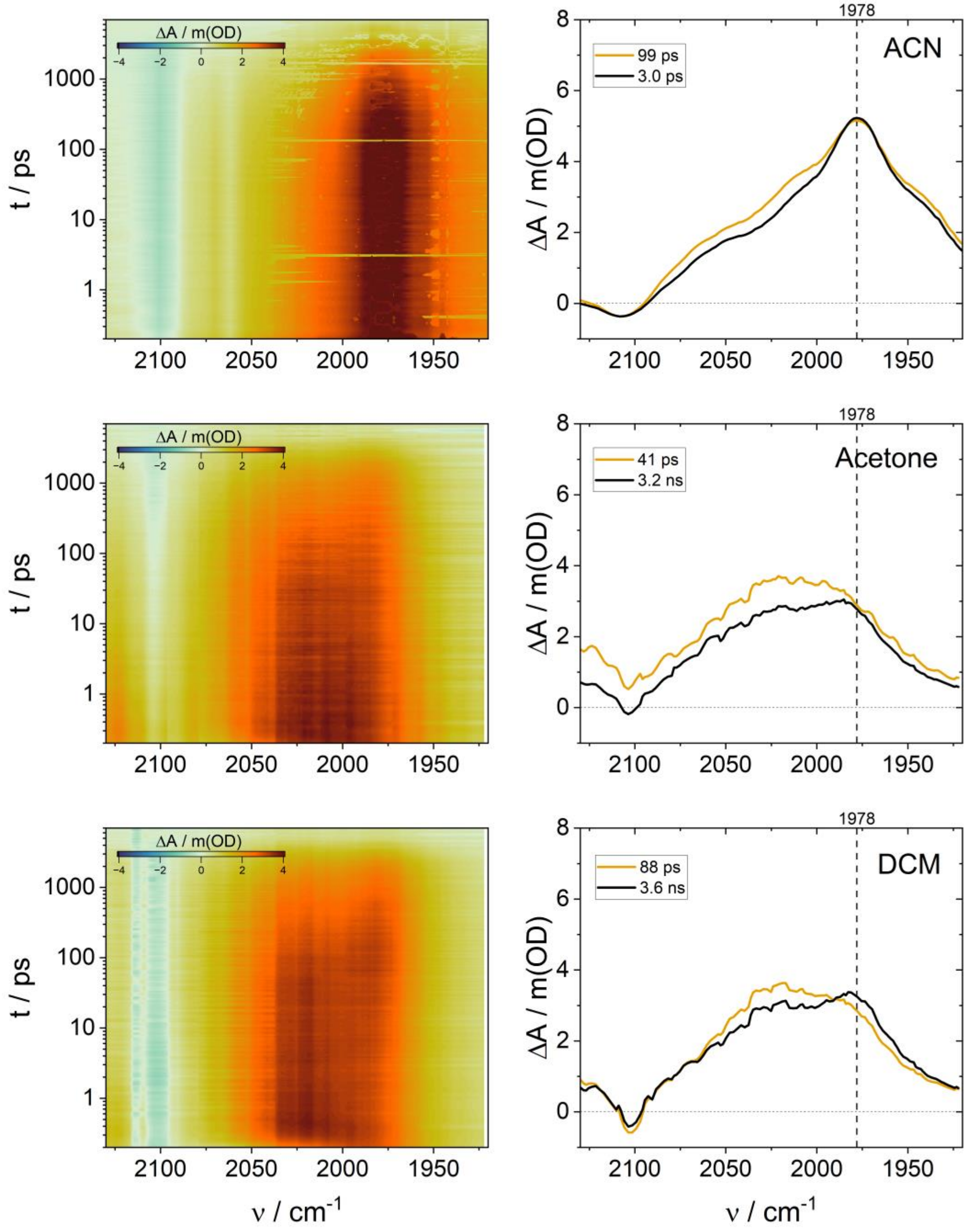

Figure S5. Heatmap (left) and species-associated spectra (right) obtained upon target analysis of fsIR measurements on RuRuDMAP in ACN (top), acetone (middle) and DCM (bottom) at room temperature.

References

[1] A. Cadranel, P. Alborés, S. Yamazaki, V. D. Kleiman, L. M. Baraldo, *Dalton Transactions* **2012**, *41*, 5343–5350.

[2] S. E. Domínguez, G. E. Pieslinger, L. Sanchez-Merlinsky, L. M. Baraldo, *Dalton Transactions* **2020**, *49*, 4125–4135.

[3] M. Krejčik, M. Daněk, F. Hartl, *J Electroanal Chem Interfacial Electrochem* **1991**, *317*, 179–187.

[4] I. H. M. Van Stokkum, D. S. Larsen, R. Van Grondelle, *Biochim Biophys Acta Bioenerg* **2004**, *1657*, 82–104.

[5] J. J. Snellenburg, S. Laptenok, R. Seger, K. M. Mullen, I. H. M. van Stokkum, *J Stat Softw* **2012**, *49*, 1–22.

[6] K. M. Mullen, I. H. M. Van Stokkum, *J Stat Softw* **2007**, *18*, 1–46.

[7] I. Ramírez-Wierzbicki, L. S. Merlinsky, G. E. Pieslinger, S. Domínguez, L. D. Slep, L. M. Baraldo, A. Cadranel, *Chemistry – A European Journal* **2024**, DOI 10.1002/chem.202402700.
